# Serum Zonulin in HBV-Associated Chronic Hepatitis, Liver Cirrhosis, and Hepatocellular Carcinoma

**DOI:** 10.1155/2019/5945721

**Published:** 2019-08-14

**Authors:** Xin Wang, Ming-ming Li, Ye Niu, Xin Zhang, Ji-bin Yin, Chang-jiu Zhao, Rui-tao Wang

**Affiliations:** ^1^Department of Internal Medicine, Harbin Medical University Cancer Hospital, Harbin Medical University, Harbin, Heilongjiang 150081, China; ^2^Department of Geriatrics, The Second Affiliated Hospital, Harbin Medical University, Harbin, Heilongjiang 150086, China; ^3^Department of Gastroenterology, The Second Affiliated Hospital, Harbin Medical University, Harbin, Heilongjiang 150086, China; ^4^Department of Nuclear Medicine, The First Affiliated Hospital of Harbin Medical University, Harbin, Heilongjiang 150001, China; ^5^Institute of Intensive Care Unit, Heilongjiang Academy of Medical Science, Harbin, Heilongjiang 150081, China

## Abstract

**Background:**

The gut microbiota is involved in the occurrence and development of chronic liver diseases. Zonulin is considered a marker of intestinal permeability. The purpose of this study was to assess zonulin levels in patients with chronic hepatitis B (CHB), HBV-associated liver cirrhosis (LC), and HBV-associated hepatocellular carcinoma (HCC).

**Materials and Methods:**

The study population consisted of 90 HBV-associated HCC patients, 90 HBV-associated LC patients, 90 CHB patients, and 90 healthy subjects. Serum levels of zonulin and AFP were determined. The diagnostic accuracy of each marker was evaluated using receiver operating characteristic (ROC) curve analysis (AUC).

**Results:**

Serum zonulin levels were significantly higher in patients with HCC than in patients with LC or CHB or healthy subjects (*p* < 0.001). Moreover, the zonulin levels were increased in the advanced stage of LC and HCC. ROC curve analysis revealed that serum zonulin could be used to differentiate CHB from cirrhosis. In addition, the combination of zonulin and AFP exhibited a significantly larger AUC compared with zonulin or AFP alone.

**Conclusions:**

Serum zonulin levels were significantly increased both in LC and in HCC and correlated with the advanced stage of LC and HCC. Moreover, the combination of zonulin and AFP confers significant benefit to diagnostic accuracy in differentiating LC from HCC.

## 1. Introduction

Chronic hepatitis B (CHB), liver cirrhosis (LC), and hepatocellular carcinoma (HCC), also known as “hepatitis trilogy”, are the three main stages in the progression of chronic liver diseases in China [1]. HCC globally ranks fifth for incidence and third for mortality among all malignant tumors. China is the most severely affected by liver cancer, which accounts for more than 50% of all HCC cases and 55% of liver cancer deaths [2]. Although some progress has been made in exploring the pathological mechanisms and interventions of chronic liver diseases, there are still no effective biomarkers for the prediction and prevention of the progression of chronic liver diseases.

Intestinal barrier is essential in many diseases such as inflammation and metabolic and autoimmune diseases [3, 4]. Tight junctions (TJ) in intestinal epithelium are important components that maintain the intestinal barrier against various environmental changes [5]. Zonulin is the only TJ-known effective physiological regulator, which is synthesized by the liver and intestines and secreted into the small intestine [6]. It is considered to be a marker of intestinal permeability since it regulates TJ to achieve reversible, rapid, and dynamic regulation of intestinal permeability. Over recent years, more and more studies have detected changes in the levels of zonulin in “intestinal leakage” diseases [4], but little is known about these same changes in different pathological conditions of the liver. Therefore, the purpose of this study was to assess zonulin levels in patients with chronic hepatitis B (CHB), HBV-associated liver cirrhosis (LC), and HBV-associated hepatocellular carcinoma (HCC).

## 2. Materials and Methods

### 2.1. Study Population

This study was a cross-sectional study. The study population consisted of 90 patients with HBV-associated HCC, 90 patients with HBV-associated liver cirrhosis (LC), 90 patients with CHB, and 90 healthy subjects. HCC patients and LC patients were admitted at the Second Affiliated Hospital of Harbin Medical University between January 2017 and December 2017. CHB patients and healthy subjects were recruited from the Second Affiliated Hospital of Harbin Medical University. They were matched for age, gender, and body mass index (BMI). The diagnosis of HCC was confirmed by histology, and none of HCC patients received any form of treatment before enrollment. Cirrhosis was diagnosed based on a biopsy or on a combination of clinical, endoscopic, and radiological evidence of portal hypertension or cirrhosis. Exclusion criteria were the following: malignant diseases other than HCC and medical treatment with corticosteroids. The clinical stage of HCC was evaluated based on the TNM classification system. Child-Pugh scoring was performed to categorize the LC and HCC patients.

The Institutional Review Board of the Second Affiliated Hospital of Harbin Medical University approved this study, and the written informed consent was obtained from all patients.

### 2.2. Clinical Examination and Biochemical Measurements

Clinical data including smoking status, drinking status, medical history, and medication use were recorded for each subject. Routine laboratory parameters were measured at the Central Laboratory of the Second Affiliated Hospital of Harbin Medical University. The inter- and intra-assay coefficients of variation (CVs) of all these assays were below 5%.

### 2.3. Blood Sampling

The blood samples from each subject were initially centrifuged at 1500g for 10 min. The serum was then aliquoted and additionally centrifuged at 2000g for 3 min at 4°C. The supernatant was then stored at -80°C.

### 2.4. ELISA Measurements

Zonulin levels were measured using a commercially available sandwich ELISA (*CUSABIO*, Wuhan, China) according to the recommendation of the manufacturer (http://www.cusabio.com). Samples were measured as duplicates. The intra- and interassay variations were below 5%.

### 2.5. Statistical Analysis

Data for continuous variables were presented using the means ± SD or median values. Data for categorical variables were presented using the frequencies and percentages. Significant differences of clinicopathological parameters among groups were determined with ANOVA, Kruskal-Wallis, or chi-square test based on the type of the data. Post hoc analyses using two-tailed LSD were conducted to compare the differences between the groups for normally distributed data. The optimal threshold values of zonulin and the overall diagnostic performance of a test were assessed by receiver operating characteristic (ROC) curve analysis. Two-sided *p* values <0.05 were considered statistically significant. Statistical analyses were performed using SPSS Statistics version 22.0 (SPSS, Inc.) and MedCalc (15.0) software.

## 3. Results

The study consisted of 90 HCC patients, 90 LC patients, 90 CHB patients, and 90 healthy subjects. Of the 360 enrolled participants, 214 (59.4%) were men and 146 (40.6%) were women. The mean ages were 53.9 ± 6.7 and 55.6 ± 7.2 years old, respectively.

**Figure 1:**
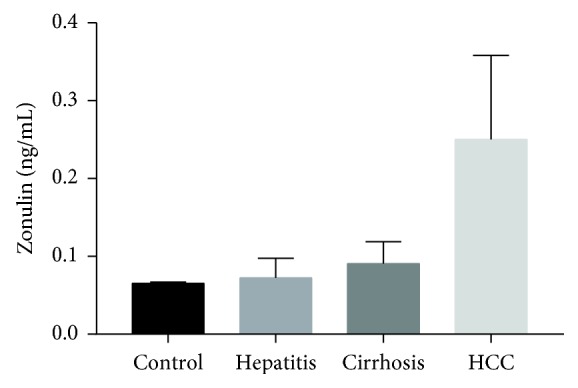
Serum zonulin levels in HCC, LC, CHB, and healthy subjects.

The clinical and laboratory characteristics of patients and control subjects are listed in Table 1. There was no difference in age, sex, BMI, smoking status, alcohol intake, and white blood cell among the four groups. However, platelet count, haemoglobin, albumin, AST, ALT, GGT, total bilirubin, and creatinine levels were significantly different among the four groups. Platelet count and albumin levels were markedly reduced, while AST, ALT, and GGT levels were increased in LC patients and HCC patients compared with CHB patients and control subjects. Haemoglobin levels were decreased, and total bilirubin levels were increased in LC patients and HCC patients compared with other groups. Notably, serum zonulin levels were significantly higher in patients with HCC than in patients with LC or CHB or healthy subjects (*p* < 0.001). Serum zonulin levels in HCC, LC, CHB, and healthy subjects were 0.250 ± 0.108, 0.091 ± 0.028, 0.072 ± 0.025, and 0.065 ± 0.002 ng/ml, respectively (Figure 1).

**Figure 2:**
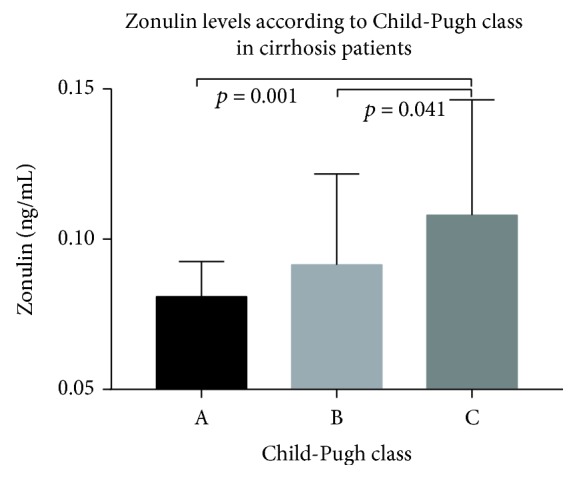
The zonulin levels according to Child-Pugh class in LC patients.

The zonulin levels according to Child-Pugh class in LC patients are shown in Figure 2. Serum zonulin levels in Child A stage, Child B stage, and Child C stage were 0.081 ± 0.012, 0.091 ± 0.030, and 0.108 ± 0.038 ng/ml, respectively. The zonulin levels were increased in Child C stage patients compared to Child A or Child B stage patients (Child C stage vs. Child A stage, *p* = 0.001; Child C stage vs. Child B stage, *p* = 0.041). However, there was no difference in zonulin levels between the patients with Child A stage and the patients with Child B stage.

**Figure 3:**
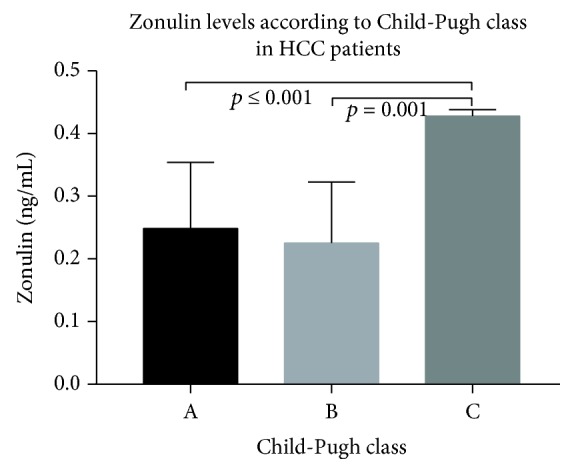
The zonulin levels according to Child-Pugh class in HCC patients.

The zonulin levels according to Child-Pugh class in HCC patients are shown in Figure 3. Serum zonulin levels in Child A stage, Child B stage, and Child C stage were 0.249 ± 0.105, 0.225 ± 0.098, and 0.428 ± 0.010 ng/ml, respectively. The zonulin levels were increased in Child C stage patients compared to Child A or Child B stage patients (Child C stage vs. Child A stage, *p* = 0.001; Child C stage vs. Child B stage, *p* = 0.001). Nevertheless, there was no difference in zonulin levels between the patients with Child A stage and the patients with Child B stage.

**Figure 4:**
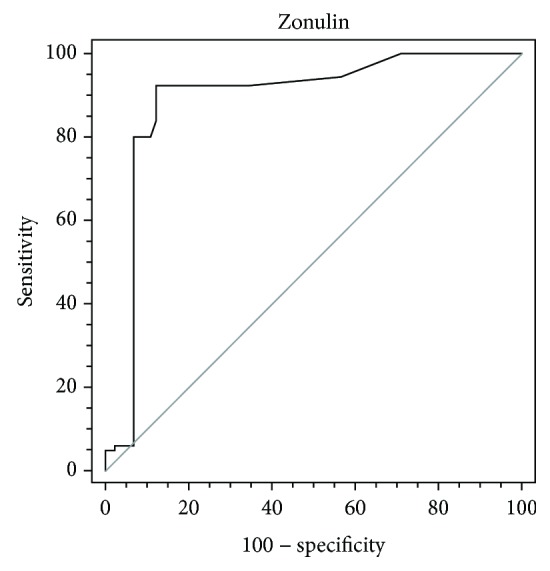
ROC curve for the utility of zonulin to differentiate CHB from LC.

**Figure 5:**
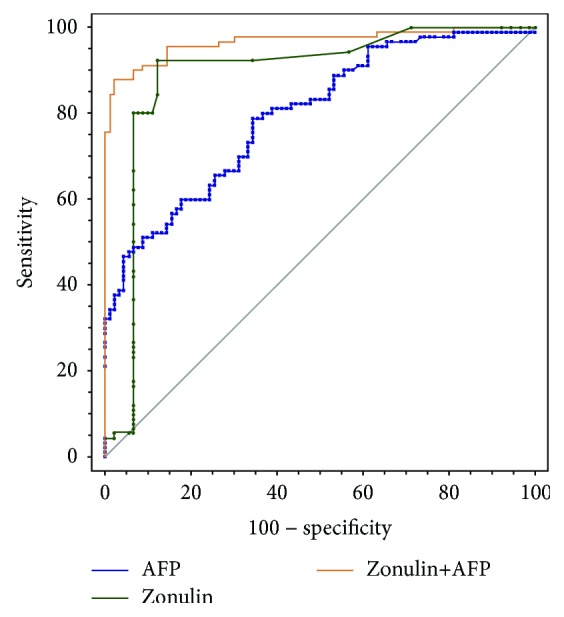
ROC curves for the utility of combined zonulin and AFP for the discrimination of LC and HCC.

To evaluate whether serum zonulin can be used as a diagnostic marker to differentiate CHB, LC, and HCC, ROC curve analysis was performed (Table 2). For the discrimination between patients with CHB and LC, zonulin had an AUC of 0.890 (95% CI 0.835-0.932), a sensitivity of 92.2%, and a specificity of 87.8%, respectively, at the cut-off value of 0.067 (Figure 4). There were 11 CHB patients and 83 LC patients whose zonulin levels were above 0.067 ng/ml. There were 79 CHB patients and 7 LC patients whose zonulin levels were equal to or less than 0.067 ng/ml. For the discrimination between patients with LC and HCC, AFP alone yielded an AUC of 0.797 (95% CI 0.931-0.854) with 78.9% sensitivity and 65.6% specificity at the cut-off value of 4.93; zonulin alone yielded an AUC of 0.942 (95% CI 0.898-0.972) with 92.2% sensitivity and 88.9% specificity at the cut-off value of 0.102. There were 31 LC patients and 71 HCC patients whose AFP levels were above 4.93 ng/ml. There were 59 LC patients and 19 HCC patients whose AFP levels were equal to or less than 4.93 ng/ml. There were 10 LC patients and 83 HCC patients whose zonulin levels were above 0.102 ng/ml. There were 80 LC patients and 7 HCC patients whose zonulin levels were equal to or less than 0.102 ng/ml. A combination of AFP and zonulin increased the specificity, but did not decrease the sensitivity. The combination of both markers exhibited a significantly larger AUC compared with AFP or zonulin (AFP vs. AFP+zonulin, *p* < 0.001; zonulin vs. AFP+zonulin, *p* = 0.013) (Figure 5).

## 4. Discussion

The main findings of the current study are the following: (1) compared with healthy control, CHB, and LC, serum zonulin was significantly increased in HCC; (2) higher levels of zonulin were correlated with advanced stage of LC and HCC; and (3) zonulin was more reliable diagnostic biomarker than the traditional marker AFP in differentiating between LC and HCC. The combination of zonulin and AFP confers significant benefit to diagnostic accuracy in differentiating LC from HCC.

The gut microbiota is involved in the occurrence and development of chronic liver diseases. The changes in the structure and abundance of gut microbiota directly affect the immune environment of the liver via “gut-liver axis” [7, 8], thereby further affecting the immune status of the body. The current study revealed that intestinal permeability significantly increased during the malignant progression of chronic liver disease. Intestinal bacterial translocation is the direct result of intestinal barrier destruction. Pathological bacteria and their harmful products (such as LPS) translocate into the portal vein, which directly exposes the liver to bacterial components that cause liver damage [9, 10] and induce the production of proinflammatory factors such as TNF-*α*, IL-6, and other immune mediators. Next, the endotoxin continuously generated by the imbalanced bacteria triggers intrahepatic and systemic inflammation, which ultimately leads to further aggravation of liver damage and dysbacteriosis, resulting in a vicious circle between the liver and the intestine [11, 12]. Xing et al. [13] have found that the number of beneficial bacteria such as Bifidobacterium and Lactobacillus significantly decreases, while the levels of harmful bacteria such as Enterobacteriaceae and Enterococcus significantly increase in CHB and LC. Wang and colleagues [14] have also reported the structural changes of gut microbiota in CHB patients. It was also observed that the structure of gut microbiota progressively changes with the development of LC and the most serious dysbacteriosis occurs in decompensated period [15]. Qin et al. [16] have shown that LC patients have severe intestinal dysbiosis compared with healthy control group, which supports the role of gut microbiome in LC. A prospective study also indicated that E. coli has a pathogenic role in the development of HCC [17]. These results are consistent with our findings according to which gut microbiota has an important role in promoting the deterioration of chronic liver disease, especially in the decompensated phase of LC and HCC.

Zonulin is a marker of intestinal permeability. It helps the intestinal barrier to cope with various physiological and pathological challenges by dynamically regulating intercellular tight junction [4, 18]. When exposed to bacteria or gluten, the small intestine can upregulate the pathological secretion of zonulin and then increase intestinal permeability and disrupt intestinal barrier function, leading to bacterial translocation and abnormal antigen presentation to the intestinal submucosa and triggering inflammation and adaptive immune response [19, 20]. This pathological process mediated by zonulin is the pathogenic mechanism for a variety of autoimmune diseases, cancers, and neurological diseases [4, 19], including inflammatory bowel disease [21], type 1 diabetes [22], and multiple sclerosis [23]. We observed a significant increase in serum zonulin levels in LC and HCC, and a dynamic change in the progression of CHB to LC and HCC, suggesting that the disruption of the intestinal barrier secondary to the disorder of zonulin pathway is a contributing factor to the deterioration of chronic liver disease. Further analysis showed that zonulin was not correlated with alanine aminotransferase, suggesting that increased zonulin may not be associated with hepatic inflammation. Consistent with our results, Raparelli et al. [24] have found that zonulin levels are higher in patients with LC, thus confirming that LC is associated with increased intestinal permeability.

There are several potential limitations to this study, including an assessment of gut microbiota and endotoxin, as well as an exploration of the molecular mechanisms between the increase in zonulin and the progression of chronic liver disease. The current study can be continued by including patients who meet the criteria to further expand the sample size and by extending the follow-up observation time so that the relationship between zonulin and chronic liver disease complications can be tracked.

In conclusion, serum zonulin levels were significantly increased in both LC and HCC and were correlated with advanced stage of LC and HCC. Moreover, the combination of zonulin and AFP confers significant benefit to diagnostic accuracy in differentiating LC from HCC. Further studies on the involvement of zonulin in LC and HCC are warranted.

## Figures and Tables

**Table 1:** Clinical and laboratory characteristics of the participants.

Variables	Healthy control	CHB	LC	HCC
Number	90	90	90	90
Age (years)	54.3 (3.7)	54.6 (3.5)	54.4 (8.2)	54.8 (10.0)
Gender (male, %)	53 (58.9)	51 (56.7)	65 (60.8)	45 (50.0)
BMI (kg/m^2^)	23.9 (2.7)	23.6 (3.2)	23.8 (3.2)	23.9 (2.9)
Current smoker (%)	28 (31.1)	30 (33.3)	20 (22.2)	30 (33.3)
Alcohol drinking (%)	32 (35.6)	33 (36.7)	30 (33.3)	24 (26.7)
WBC (×10^9^/l)	5.8 (1.3)	5.9 (1.4)	6.3 (3.7)	5.5 (2.4)
Platelet count (×10^9^/l)	223.0 (57.1)	224.1 (61.7)	112.9 (64.6)	140.4 (74.6)
Haemoglobin (g/l)	147.9 (14.7)	150.2 (12.7)	108.1 (35.2)	135.5 (17.0)
Albumin (g/l)	47.0 (2.1)	47.1 (2.5)	33.3 (7.7)	37.4 (6.9)
AST (U/l)	23.0 (18.0-31.0)	21.0 (15.8-31.0)	46.0 (26.0-73.0)	21.8 (21.8-69.5)
ALT (U/l)	20.5 (15.0-33.3)	23.0 (14.8-34.0)	33.0 (22.0-66.5)	32.0 (19.0-65.6)
GGT (U/l)	22.5 (13.0-41.3)	28.0 (16.5-49.3)	47.0 (27.0-127.0)	70.4 (40.7-141.0)
TB (*μ*mol/l)	9.0 (7.7-17.2)	15.6 (8.3-20.4)	30.5 (15.3-51.3)	14.9 (10.4-28.0)
Creatinine (*μ*mol/l)	72.5 (14.1)	71.1 (13.4)	86.8 (47.7)	70.9 (13.7)
AFP (ng/ml)	—	0.94 (0.83-2.89)	3.49 (1.90-13.28)	38.71 (5.32-887.00)
Child-Pugh class				
A	—	—	35 (38.9)	61 (67.8)
B	—	—	38 (42.2)	25 (27.8)
C	—	—	16 (17.8)	4 (4.4)
Tumor size (cm)				
≥5 cm	—	—	—	46 (51.1)
<5 cm	—	—	—	44 (48.9)
Tumor number				
Single	—	—	—	74 (82.2)
Multiple	—	—	—	16 (17.8)
Tumor differentiation				
Well/moderately	—	—	—	64 (71.1)
Poorly	—	—	—	26 (28.9)
Tumor stage				
I-II	—	—	—	41 (45.6)
III-IV	—	—	—	49 (54.4)

Data are presented as means (SD) or median (interquartile range) or percentage. BMI: body mass index; WBC: white blood cells; AST: aspartate aminotransferase; ALT: alanine aminotransferase; GGT: gamma-glutamyl transpeptidase; TB: total bilirubin; AFP: *α*-fetoprotein; CHB: chronic hepatitis B; LC: HBV-associated liver cirrhosis; HCC: HBV-associated hepatocellular carcinoma.

**Table 2:** Receiver operating characteristic curve analyses showing the utility of alone or combined markers for differentiating of LC and HCC.

Marker	Sensitivity	Specificity	PPV	NPV	AUC
Zonulin (ng/ml)	0.922	0.889	0.892	0.920	0.942 (0.898-0.972)
AFP (ng/ml)	0.789	0.656	0.696	0.756	0.797 (0.731-0.854)
Zonulin+AFP	0.878	0.978	0.878	0.978	0.967 (0.929-0.988)

PPV: positive predictive value; NPV: negative predictive value; AUC: area under the curve.

## Data Availability

The data used to support the findings of this study are available from the corresponding author upon request.
